# Trm5 and TrmD: Two Enzymes from Distinct Origins Catalyze the Identical tRNA Modification, m^1^G37

**DOI:** 10.3390/biom7010032

**Published:** 2017-03-21

**Authors:** Sakurako Goto-Ito, Takuhiro Ito, Shigeyuki Yokoyama

**Affiliations:** 1 Institute of Molecular and Cellular Biosciences, The University of Tokyo, Tokyo 113-0032, Japan; sakurako@iam.u-tokyo.ac.jp; 2 Division of Structural and Synthetic Biology, RIKEN Center for Life Science Technologies, Yokohama 230-0045, Japan; 3 RIKEN Structural Biology Laboratory, Yokohama 230-0045, Japan

**Keywords:** m^1^G37, Trm5, TrmD, tRNA

## Abstract

The *N*^1^-atom of guanosine at position 37 in transfer RNA (tRNA) is methylated by tRNA methyltransferase 5 (Trm5) in eukaryotes and archaea, and by tRNA methyltransferase D (TrmD) in bacteria. The resultant modified nucleotide m^1^G37 positively regulates the aminoacylation of the tRNA, and simultaneously functions to prevent the +1 frameshift on the ribosome. Interestingly, Trm5 and TrmD have completely distinct origins, and therefore bear different tertiary folds. In this review, we describe the different strategies utilized by Trm5 and TrmD to recognize their substrate tRNAs, mainly based on their crystal structures complexed with substrate tRNAs.

## 1. Introduction

Over one hundred kinds of nucleotide modifications have been found in transfer RNAs (tRNAs) [1]. The functions of the nucleotide modifications in tRNAs can be roughly divided into two categories: the stabilization of the functional L-shape and the maintenance of the accuracy during the decoding process [2,3,4]. Many of the modifications for the latter function are targeted to position 34, the wobble nucleotide, and position 37, the nucleotide 3′-adjacent to the anticodon. Among them, the *N*^1^-methylguanosine at position 37 (m^1^G37) is conserved among all three domains of life. The well-known function of m^1^G37 is the prevention of the +1 frameshift during translation on the ribosome. When a slippery sequence (e.g., CCCC) is present in the messenger RNA (mRNA), the absence of the m^1^G37 modification of the complementary tRNA^Pro^ increases the rate of the +1 frameshift [5,6,7]. Another important function of m^1^G37 is the regulation of effective aminoacylation. m^1^G37 in archaeal tRNA^Cys^_GCA_ promotes its aminoacylation rates of phosphoseryl-tRNA synthetase and cysteinyl-tRNA synthetase [8,9]. Additionally, m^1^G37 in yeast tRNA^Asp^_GUC_ prevents the incorrect aminoacylation with arginine by arginyl-tRNA synthetase [10]. Consequently, the deficiency of the m^1^G37 modification results in severe cell growth defects [5,11,12,13,14]. 

The importance of the m^1^G37 modification is common in the three domains of life. However, depending on the domain, the m^1^G37 modification is performed by two different enzymes: tRNA methyltransferase 5 (Trm5) in eukaryotes and archaea, and tRNA methyltransferase D (TrmD) in bacteria. Although both enzymes use *S*-adenosyl-L-methionine (AdoMet) as a methyl donor, they have distinct origins and bear different tertiary folds. Trm5 belongs to the most common class of methyltransferases (MTases): class I, which utilizes a Rossmann-fold for the AdoMet binding. TrmD belongs to class IV or the SPOUT class, named after SpoU and TrmD. The MTases in the SPOUT class possess a deep trefoil knot—which is quite exceptional for proteins—at the C-terminal region of the protein. TrmD requires not only G37 but also G36 as a substrate tRNA sequence, and the nine-base pair RNA duplex consisting of the anticodon and D stems with the anticodon loop can serve as its minimum substrate [15,16]. In contrast, Trm5 requires G37 together with the entire tRNA structure [15,16]. The characteristics of Trm5 and TrmD are summarized in Table 1. 

Phylogenetic analyses revealed that the archaeal Trm5s can be classified into three categories: Trm5a, Trm5b, and Trm5c [17]. Trm5b is found in most euryarchaea, and is considered to be the original Trm5 in euryarchaea. Trm5a exists in all crenarchaea examined thus far, and thus probably originated in crenarchaea [17]. Some euryarchaea have Trm5a as well as Trm5b. Furthermore, two crenarchaeal orders have Trm5c, which is proposed to have originated from euryarchaea by horizontal gene transfer. Trm5a, Trm5b, and Trm5c all perform the *N^1^*-methylation of tRNA G37. Curiously, in addition to the methylation of the *N*^1^-atom of guanosine, Trm5a catalyzes the methylation of the *C*^7^-atom of 4-demethylwyosine, which is the intermediate of the wyosine derivatives found at position 37 of archaeal tRNA^Phe^.

## 2. Trm5

### 2.1. Trm5 Structure

In 2008, our group reported the crystal structure of *Methanocaldococcus jannaschii* archaeal Trm5 in complex with the AdoMet analog sinefungin [18]. *M. jannaschii* Trm5 belongs to Trm5b. This structure revealed that Trm5 consists of three structural domains: domain 1 (D1), domain 2 (D2), and domain 3 (D3). D1 corresponds to the less-conserved region among Trm5 enzymes from all species, while D2 corresponds to the conserved region. The structure of D2 shares homology with that of TYW2, the tRNA-wybutosine (yW) synthesizing enzyme-2 [19]. D3 corresponds to the Rossmann-fold domain containing the AdoMet binding site, and is conserved among the class-I MTases. A kinetic analysis at 50 °C revealed that the D2–D3 fragment alone possesses methyl-transfer activity comparable to that of the full-length enzyme, although the presence of D1 lowers and enhances the *K*_M_ and *k*_cat_ values (the Michaelis and catalytic rate constants, respectively, in the Michaelis–Menten equation) for tRNA, respectively, as compared to the D2–D3 fragment. Furthermore, the nuclear magnetic resonance (NMR) analysis suggested that D1 is connected to D2 by a flexible loop and behaves almost independently of the other domains. 

Recently, the structures of Trm5a from *Pyrococcus abyssi* were determined in complex with several AdoMet analogues [20]. The structures of *P. abyssi* D1 and D2-D3 are similar to those of *M. jannaschii* Trm5. Similar to *M. jannaschii* Trm5, D1 of *P. abyssi* Trm5a behaves independently from D2–D3, as suggested by the fluorescence resonance energy transfer (FRET) analysis. 

### 2.2. tRNA Recognition by Trm5

**Figure 1:**
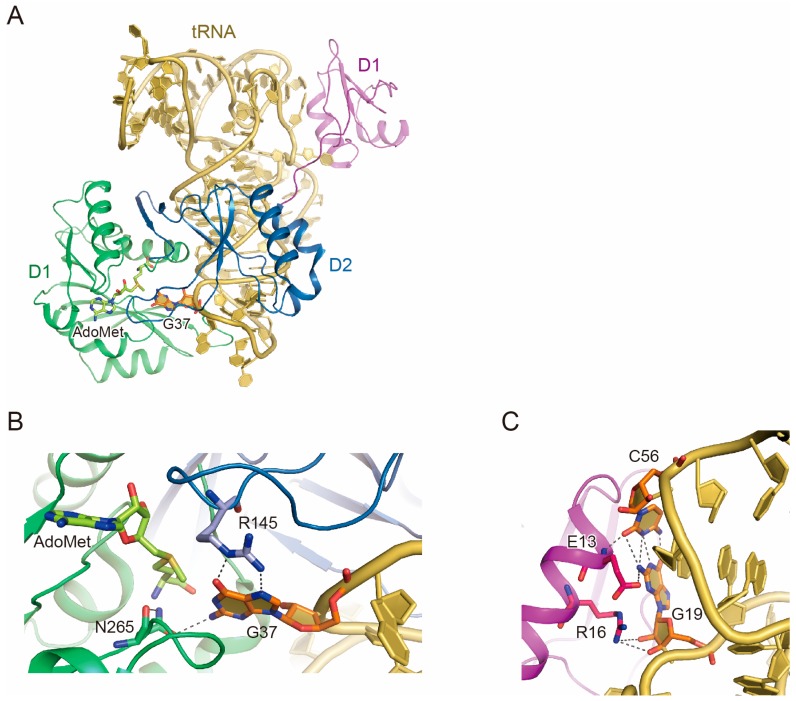
Crystal structure of *Methanocaldococcus jannaschii* Trm5·tRNA·AdoMet complex. (**A**) Overall structure of the *M. jannaschii* Trm5·tRNA·AdoMet complex. In Trm5, domains D1, D2, and D3 are colored violet, blue, and green, respectively. The tRNA is colored beige, with G37 highlighted in orange. The AdoMet molecule is shown by a yellow-green stick model; (**B**) G37 recognition by Trm5. The interacting residues of Trm5 and G37 are represented by stick models. The hydrogen bonds between Trm5 and G37 are indicated by grey dotted lines; (**C**) Recognition of the G19:C56 tertiary base pair by Trm5. The interacting residues of Trm5 and G19:C56 are represented by stick models. The hydrogen bonds between Trm5 and G19:C56 are indicated by grey dotted lines.

Subsequently, our group reported the crystal structures of the *M. jannaschii* Trm5·AdoMet·tRNA complexes with two kinds of tRNA transcripts [21]. In these complex structures, the catalytic domains of D2 and D3 interact with the tRNA anticodon branch, whereas D1 interacts with the outer corner of the L-shaped tRNA, where the D and T loops are clamped (Figure 1a). 

The structure of positions 33–37 in the anticodon loop is largely altered from the canonical tRNA structure, and the target G37 is flipped out into the catalytic pocket formed by the D2 and D3 domains. The flipped G37 is recognized in a guanosine-specific manner by the side chains of Arg145 and Asn265, and the *N*^1^-atom (the methylation atom) of G37 is located next to the methyl moiety of AdoMet (Figure 1b). Asn265 is the N-terminal residue of the NPPY ([D/N/S][I/P]P[Y/F/W/H]) motif, which is conserved among the class-I amino-methyl transferases. Interestingly, *P. abyssi* Trm5a harbors the Pro260-Thr261-Pro262-Lys263 sequence at the corresponding position. The superimposition of the *P. abyssi* Trm5a·*S*-adenosyl-L-homocysteine structure on the *M. jannaschii* Trm5·AdoMet·tRNA structure indicated that Pro260 is too far away to contact the *N*^1^-atom of G37. The broad substrate recognition pocket may accommodate substrate tRNAs with 4-demethylwyosine at position 37. The backbone phosphate groups at positions 38–41 in the anticodon arm and those at positions 24–26 in the D stem—which form the frame of the major groove of the tRNA structure—interact with D2 and D3 of Trm5. The bases of C32 and A38 face each other and stack with Pro322, which may be a key interaction for Trm5 to find position 37. Along with biochemical assays, the structure indicated that the D2–D3 domains contain all of the elements required for the specific positioning of G37 and the *N*^1^-methylation of the guanine base. 

### 2.3. Function of D1

We examined the function of D1. In our crystal structures, D1 directly recognizes the outer corner of the L-shaped tRNA (Figure 1a). At the outer corner of the L-shape, G19 from the D loop and C56 from the T loop form the tertiary interloop base pairing, which is conserved among tRNAs. As shown in Figure 1c, the G19:C56 base pair is specifically recognized by Trm5. We thus analyzed the significance of D1 by tRNA methylation assays with the wild-type and mutant forms of Trm5 and tRNA. Interestingly, increasing the reaction temperature to 60–70 °C reduced the activity of the D2–D3 fragment, but enhanced that of the full-length Trm5. Furthermore, increasing the temperature up to 80 °C diminished the activity of the D2–D3 fragment, but that of the full-length Trm5 remained. Therefore, D1 plays an important role in the methylation at high temperature. Experiments using the Trm5 mutants with alanine substitutions of the D1 residues that interact with the outer corner of the tRNA or the tRNA mutants that disrupt the G19:C56 base pair revealed that the interaction between Trm5 D1 and the outer corner of the tRNA is responsible for the activity at high temperature. In addition, kinetic experiments demonstrated that the tRNA mutations that disrupt the G19:C56 base pair reduced the activity of full-length Trm5 at 70 °C by enhancing the *K*_M_ values but maintaining the *k*_cat_ values. Likewise, the Trm5 mutant with alanine substitutions of the D1 residues that interact with the tRNA outer corner had a higher *K*_M_ value than the wild-type Trm5. These results suggested that the interaction between the outer-corner of the tRNA and Trm5 D1 is essential to confer sufficiently robust affinity for the tRNA at physiological temperatures. 

Although the overall structures of D1 are similar between *M. jannaschii* Trm5b and *P. abyssi* Trm5a, the *M. jannaschii* Trm5b residues involved in the G19:C56 recognition are not conserved in *P. abyssi* Trm5a. The residues corresponding to Glu13 and Arg16 in *M. jannaschii* Trm5b are Lys12 and Leu15 in *P. abyssi* Trm5a, respectively. Further investigations are required to reveal the function of Trm5a D1, as it may have a distinct tRNA recognition mechanism or it may not function in tRNA recognition. 

### 2.4. tRNA Maturation Check by Trm5

tRNAs contain various modifications in the buried regions of the L-shape. For example, ArcTGT introduces archaeosine (7-formamidino-7-deazaguanosine) at position 15 in the D-loop, and archaeosine contributes to the stabilization of the L-shaped tRNA [22]. The crystal structure of ArcTGT complexed with the substrate tRNA revealed that the tRNA assumed an alternative conformation called the λ-form [23]. In the λ-form, G19 and C56 are distant from each other. Apparently, the G19:C56 base pair is formed only after the L-shape is correctly formed. The L-shapes of tRNA transcripts are unstable compared to the fully-modified tRNAs [24,25]. Especially the G19:C56 base pair is often disrupted in tRNA transcripts, but stable in the fully-modified tRNAs [26]. 

As explained above, the crystal structures and the subsequent biochemical assays demonstrated that D1 recognizes the proper conformation of the outer corner of the L-shaped tRNA, in which the hydrogen-bonded G19:C56 base pair is stabilized. The adequate interaction between D1 and tRNA enables the catalytic D2-D3 to perform the m^1^G37 methylation. In other words, the m^1^G37 methylation is achieved by a “sensor–effector” mechanism in which the affinity of Trm5 for tRNA increases only when the sensor (D1) confirms the completion of the L-shape formation and the catalytically competent “effector” (D2-D3) is recruited to the tRNA. The completion of the L-shape formation with the hydrogen-bonded G19:C56 pair is accomplished by the completion of the nucleotide modifications buried in the tRNA, such as archaeosine at position 15 and pseudouridine at position 55. Once m^1^G37 is formed, the tRNA starts functioning in translation, as m^1^G37 enhances the aminoacylation and ribosomal entry rates and concomitantly prevents frameshifts and incorrect aminoacylation. Despite the importance of D1 (as revealed in *M. jannaschii* Trm5), some crenarchaeal Trm5a molecules lack D1. Several possibilities remain to be verified. Different molecules may be responsible for the tRNA maturation check, or the tRNA maturation check may be unnecessary in some organisms due to their environment. 

## 3. TrmD

### 3.1. TrmD Structure

The crystal structures of the TrmDs from *Haemophilus influenzae* and *Escherichia coli* were reported from two groups in 2003 [27,28]. TrmD consists of the N-terminal domain (NTD, the SPOUT domain) and the TrmD-specific C-terminal domain (CTD). These domains are connected by the interdomain linker. TrmD forms a homodimer, and the interdomain linkers are disordered in both monomers. The trefoil knot at the C-terminal region in the SPOUT domain provides the AdoMet-binding site.

### 3.2. The Binding of the TrmD Dimer to a Substrate tRNA

**Figure 2:**
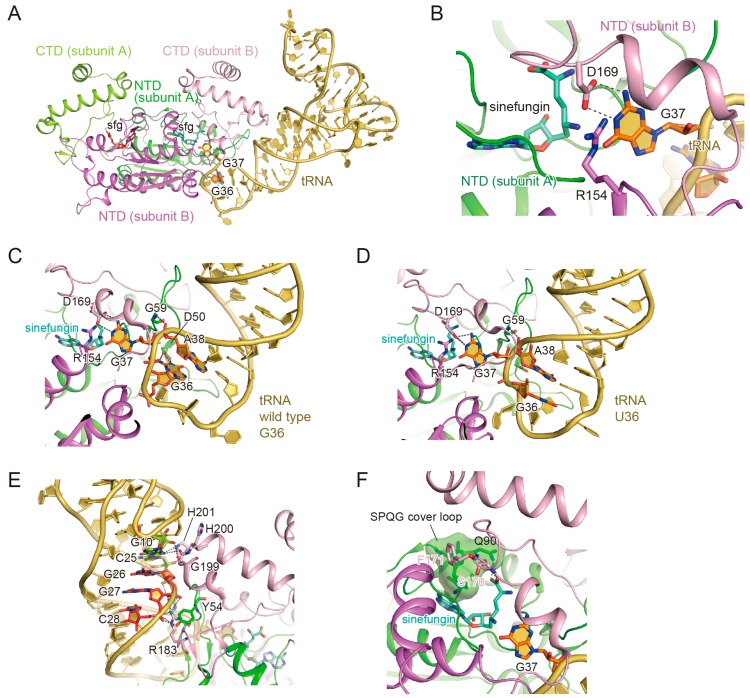
Crystal structure of *Haemophilus influenzae* TrmD·tRNA·sinefungin complex. (**A**) Overall structure of the *H. influenzae* TrmD·tRNA·sinefungin complex. In TrmD, the N-terminal domain (NTD) of subunit A, the C-terminal domain (CTD) of subunit A, the NTD of subunit B, and the CTD of subunit B are colored green, yellow-green, violet, and light pink, respectively. The tRNA is colored beige, with G36 and G37 highlighted in orange. Sinefungin molecules bound to subunit A and subunit B are shown in stick models colored pale green and brown, respectively; (**B**) G37 recognition by TrmD. The interacting residues of TrmD and G37 are represented by stick models. The hydrogen bonds between TrmD and G37 are indicated by grey dotted lines; (**C**,**D**) The anticodon recognition in the TrmD·tRNA (G36 in (C), U36 in (D))·sinefungin complex. The residues involved in the recognition are shown in stick models. The hydrogen bonds between TrmD and G37 are indicated by grey dotted lines; (**E**) D- and anticodon-stem recognition by TrmD. The residues involved in the recognition are shown in stick models. The hydrogen bonds between TrmD and tRNA are indicated by grey dotted lines; (**F**) Methyl-donor recognition by TrmD. The SPQG (Ser-Pro-Gln-Gly) cover loop is indicated in a surface representation. The residues involved in the recognition are shown in stick models. The hydrogen bonds between TrmD and sinefungin are indicated by grey dotted lines.

In 2015, our group reported the crystal structure of the *H. influenzae* TrmD·tRNA·sinefungin complex [29]. In this complex structure, one TrmD homodimer is complexed with one tRNA molecule, and each TrmD monomer binds a sinefungin molecule (Figure 2a). This binding mode is consistent with the previously reported biochemical analysis [30]. When the substrate tRNA interacts with the catalytic pocket of subunit A, the NTDs of subunits A and B, and the neighboring CTD of subunit B, contact the tRNA (Figure 2a). The interdomain linker of subunit B forms a helix upon tRNA binding, and participates in the interaction with the substrate tRNA.

### 3.3. The Interaction with G37

The base moiety of G37 is flipped out from the anticodon loop, and protrudes into the catalytic pocket. The 1-NH and 2-NH2 groups of G37 hydrogen bond with the side-chain of Asp169 in subunit B, which makes the recognition guanosine-specific (Figure 2b). The side-chain of Arg154 in subunit B is located next to the guanosine base of G37. The ε amino group of sinefungin—corresponding to the donor methyl group of AdoMet—is located next to the 1-NH group. Since the alanine substitutions of Arg154 and Asp169 substantially decreased the methyl transfer activities of TrmD [29], these two residues play fundamental roles in the catalysis of methyl transfer. Asp169 is likely to accept a proton from the 1-NH of G37, and Arg154 may stabilize the resultant deprotonated intermediate state until the methyl transfer reaction, consistent with the previous biochemical studies [28].

### 3.4. The Interaction with G36

The requirement of G36 and G37 has been well studied in TrmD [15,16]. In our TrmD–tRNA complex structure, G36 actually interacts guanosine-specifically with the side-chain carboxyl group of Asp50, which is strictly conserved in TrmD (Figure 2c). The guanosine base of G36 is stabilized in the *syn* conformation, which is rare in tRNA molecules. This G36-interacting pocket is formed only after the guanosine base of G37 is flipped from the anticodon loop and captured by Asp169 and Arg154, indicating that the G36 interaction occurs only after the G37 interaction. 

In order to further investigate the cooperativity of the recognitions of G36 and G37, we determined the crystal structures of TrmD·sinefungin in complex with the G36U or G36C tRNA variant. In these mutant structures, the pyrimidine base at position 36 does not interact with Asp50, and is directed toward the outside of the anticodon loop (Figure 2d), consistent with our kinetic experiments using the tRNA variants mutated at position 36 [29]. In addition, while the main-chain NH group of the strictly conserved Gly59 hydrogen bonds with the O^2P^ atom of A38 in the complex with the wild-type tRNA, the same NH group hydrogen bonds with the O^1P^ atom of A38 in the complex with the variant tRNA. The presence of guanosine at position 36 thereby induces the conformational change in the tRNA anticodon loop and stabilizes it in the “tight” form, as compared to the “loose” form in the absence of guanosine at position 36. Interestingly, the coordinates of the G37 base are quite similar among the determined structures, consistent with the hypothesis that the G37 binding by TrmD needs to precede the G36 interaction. Moreover, the absence of guanosine at position 36 dramatically changes the conformation of the interdomain linker of TrmD. The linker folds into a helix when it is bound to the wild-type tRNA, while it is disordered when bound to the variant tRNAs. This probably occurs because the “loose” anticodon loop conformation in the tRNA variants narrows the space for the interdomain helix to fold over the G37-binding pocket. These conformational changes in TrmD and tRNA following the G37 recognition—especially the G36 recognition and the interdomain helix folding—further strengthen the G37 recognition and make it suitable for the methyl transfer reaction.

### 3.5. Anticodon-Branch Recognition and Detection of Position 37

How does TrmD search for position 37 in the substrate tRNA? First, the CTD of subunit B binds to the D-stem in the following manner. The conserved Ser198-Gly199-His/Asp200-His201 residues interact with the G10:C25 base pair, which is basically conserved in the substrate tRNAs (Figure 2e). Second, the phosphate groups of G26, G27, and C28 on the 5′ strand of the anticodon arm hydrogen bond with Arg52 of subunit A, Tyr54 of subunit A, Arg183 of subunit B, and several main chain atoms in the NTD of subunit A or the CTD of subunit B. Many hydrogen bonds are formed between these phosphate groups and the TrmD residues, indicating the rigid interaction of this region. Finally, in the anticodon loop, the phosphate group of A38—3′-adjacent to position 37—hydrogen bonds with the main-chain NH group of Gly59 in the NTD of subunit A, as mentioned above. This interaction manner elegantly explains why TrmD requires only the nine-base pair RNA duplex with the central anticodon loop [15,16].

### 3.6. Structural Changes of TrmD upon AdoMet Accommodation

Through detailed analyses of various TrmD structures (including the apo, adenosine-bound [31], AdoMet-bound, and tRNA·sinefungin-bound forms), we discovered the structural changes accompanying AdoMet binding. The significant difference is the orientation of Phe171 in subunit B. When TrmD binds AdoMet or sinefungin, the sidechain of Phe171 in subunit B protrudes between the CTD of subunit B and the NTD of subunit A (Figure 2f). In contrast, Phe171 is disordered in the apo and adenosine-bound TrmD structures. The protruding Phe171 of subunit B places the adjacent Ser170 within hydrogen-bonding distance with the carboxy moiety of the methionine in AdoMet and Gln90 in subunit A, which also recognizes the carboxy moiety of AdoMet. Consistently, the alanine substitution of Phe171 completely abolished the TrmD activity [28], and the structure precisely explains the importance of Phe171. 

Next, we investigated how TrmD binds the AdoMet molecule. The adenosine moiety of AdoMet is buried inside the deep trefoil knot and is covered by the ^88^SPQG^91^ residues in the middle of the deep trefoil knot structure. Accordingly, the structural change of the loop including the ^88^SPQG^91^ residues—hereafter called the “cover loop”—is required to accommodate the AdoMet molecule. The high B-factors of the ^88^SPQG^91^ residues and the residues surrounding the cover loop indicate the structural flexibility of this region. Therefore, we propose that the cover loop can assume the open conformation, which allows AdoMet to enter its binding site. In all of the SPOUT domain structures determined thus far, the B factors of the cover loop are higher than those of the other residues forming the deep trefoil knot [29]. Therefore, the mechanism of AdoMet accommodation using the cover loop is widely adopted among the SPOUT methyltransferase family members.

### 3.7. Enzymatic Cycle of TrmD

Based on our findings, we proposed a model for the TrmD enzymatic cycle which consists of the AdoMet-binding, tRNA-binding, and methyl transfer stages.

1. AdoMet binding: In apo TrmD, the closed and open conformations of the cover loop form the “tight-knot” and “loose-knot” states, respectively, and AdoMet can be accommodated only in the “loose-knot” state. Subsequently, the bound AdoMet is stabilized by Phe171, which also allows the CTD to assume the tRNA binding form.
2. tRNA binding: Following AdoMet binding, TrmD starts searching for position 37 in the substrate tRNA. Considering the steric hindrance, we propose that TrmD first binds to the phosphate groups in the anticodon stem. Then, TrmD and the tRNA anticodon loop mutually change their conformations, and finally, Gly59 captures the phosphate group at position 38. This Gly59–position 38 interaction flips the base moiety of position 37 into the catalytic pocket. TrmD then binds tightly to the tRNA when position 37 is guanosine. In the anticodon loop with G37 trapped, TrmD further recognizes G36 in a guanosine-specific manner. After the recognition of both G36 and G37 by TrmD, the anticodon loop structure adopts the tight form, which provides space for the TrmD interdomain helix to fold above G37.
3. Methyl transfer: At this point, the substrate tRNA is tightly trapped by TrmD, and the arrangements of the AdoMet, G37, and the residues composing the catalytic site are ready for the reaction. The methyl moiety of AdoMet is transferred to the *N*^1^-atom of G37, producing the m^1^G37-modified tRNA and the byproduct, AdoHcy. Upon the collapse of the interdomain helix and the opening of the cover loop, the reaction products are released from TrmD, which makes TrmD ready for the next reaction.

## 4. Conclusions

In this review, we described how TrmD and Trm5 find a substrate tRNA to transfer the methyl moiety of AdoMet to the *N*^1^-atom of G37, based on their crystal structures in complex with the substrate tRNAs and AdoMet or its analog. Although their strategies are quite different, they both have additional requirements that are necessary to stably bind tRNAs: Trm5 requires the stable base pair between G19 and C56 with the L-shaped tRNA, while TrmD requires G36 with the structure of the D stem and the anticodon arm. These requirements may have worked beneficially during the long evolution of life by improving the translational fidelity and efficiency. However, the reason why these enzymes with distinct origins perform identical reactions still remains to be elucidated. Further analyses, such as investigations of unidentified binding partners, may provide insights to answer this interesting question. 

## Figures and Tables

**Table 1:** The characteristics of transfer RNA (tRNA) methyltransferase 5 (Trm5) and tRNA methyltransferase D (TrmD).

	Trm5	TrmD
**Reaction catalyzed**	*N*^1^-methylation of tRNA G37	*N*^1^-methylation of tRNA G37
**Organisms**	Eukaryotes, Archaea	Bacteria
**MTase class**	Class-I	Class-IV
**Protein fold**	Rossmann fold, monomer	Deep-trefoil knot, dimer
**Cofactor**	AdoMet	AdoMet
**Substrate requirement**	L-shaped tRNA with G37	tRNA anticodon stem loop with G36G37 and D stem
**Stoichiometry**	1 tRNA/1 Trm5	1 tRNA/2 TrmD

AdoMet: *S*-adenosyl-L-methionine; MTase: Methyltransferase.

## References

[1] Czerwoniec A., Dunin-Horkawicz S., Purta E., Kaminska K.H., Kasprzak J.M., Bujnicki J.M., Grosjean H., Rother K. (2009). Modomics: A database of RNA modification pathways. Nucleic Acids Res..

[2] Yokoyama S., Nishimura S., Söll D., Rajbhandary U.L. (1995). Modified nucleotides and codon recognition. tRNA: Structure, Biosynthesis, and Function.

[3] Bjork G.R., Söll D., Rajbhandary U.L. (1995). Biosynthesis and function of modified nucleotides. tRNA: Structure, Biosynthesis, and Function.

[4] Helm M. (2006). Post-transcriptional nucleotide modification and alternative folding of RNA. Nucleic Acids Res..

[5] Bjork G.R., Wikstrom P.M., Bystrom A.S. (1989). Prevention of translational frameshifting by the modified nucleoside 1-methylguanosine. Science.

[6] Hagervall T.G., Tuohy T.M., Atkins J.F., Bjork G.R. (1993). Deficiency of 1-methylguanosine in tRNA from *Salmonella typhimurium* induces frameshifting by quadruplet translocation. J. Mol. Biol..

[7] Gamper H.B., Masuda I., Frenkel-Morgenstern M., Hou Y.M. (2015). Maintenance of protein synthesis reading frame by EF-P and m^1^G37-tRNA. Nat. Commun..

[8] Zhang C.M., Liu C., Slater S., Hou Y.M. (2008). Aminoacylation of tRNA with phosphoserine for synthesis of cysteinyl-tRNA^cys^. Nat. Struct. Mol. Biol..

[9] Hauenstein S.I., Perona J.J. (2008). Redundant synthesis of cysteinyl-tRNA^cys^ in *Methanosarcina mazei*. J. Biol. Chem..

[10] Perret V., Garcia A., Grosjean H., Ebel J.P., Florentz C., Giege R. (1990). Relaxation of a transfer RNA specificity by removal of modified nucleotides. Nature.

[11] Bystrom A.S., Bjork G.R. (1982). Chromosomal location and cloning of the gene (trmD) responsible for the synthesis of tRNA m^1^G methyltransferase in *Escherichia coli* k-12. Mol. Gen. Genet. MGG.

[12] Urbonavicius J., Qian Q., Durand J.M., Hagervall T.G., Bjork G.R. (2001). Improvement of reading frame maintenance is a common function for several tRNA modifications. EMBO J..

[13] Persson B.C., Bylund G.O., Berg D.E., Wikstrom P.M. (1995). Functional analysis of the ffh-trmD region of the *Escherichia coli* chromosome by using reverse genetics. J. Bacteriol..

[14] Li J., Esberg B., Curran J.F., Bjork G.R. (1997). Three modified nucleosides present in the anticodon stem and loop influence the in vivo aa-tRNA selection in a tRNA-dependent manner. J. Mol. Biol..

[15] Brule H., Elliott M., Redlak M., Zehner Z.E., Holmes W.M. (2004). Isolation and characterization of the human tRNA-(N^1^G37) methyltransferase (TRM5) and comparison to the *Escherichia coli* TrmD protein. Biochemistry.

[16] Christian T., Hou Y.M. (2007). Distinct determinants of tRNA recognition by the TrmD and Trm5 methyl transferases. J. Mol. Biol..

[17] De Crecy-Lagard V., Brochier-Armanet C., Urbonavicius J., Fernandez B., Phillips G., Lyons B., Noma A., Alvarez S., Droogmans L., Armengaud J. (2010). Biosynthesis of wyosine derivatives in tRNA: An ancient and highly diverse pathway in archaea. Mol. Biol. Evol..

[18] Goto-Ito S., Ito T., Ishii R., Muto Y., Bessho Y., Yokoyama S. (2008). Crystal structure of archaeal tRNA(m^1^G37)methyltransferase aTrm5. Proteins.

[19] Umitsu M., Nishimasu H., Noma A., Suzuki T., Ishitani R., Nureki O. (2009). Structural basis of adomet-dependent aminocarboxypropyl transfer reaction catalyzed by tRNA-wybutosine synthesizing enzyme, TYW2. Proc. Natl. Acad. Sci. USA.

[20] Wang C., Jia Q., Chen R., Wei Y., Li J., Ma J., Xie W. (2016). Crystal structures of the bifunctional tRNA methyltransferase Trm5a. Sci. Rep..

[21] Goto-Ito S., Ito T., Kuratani M., Bessho Y., Yokoyama S. (2009). Tertiary structure checkpoint at anticodon loop modification in tRNA functional maturation. Nat. Struct. Mol. Biol..

[22] Watanabe M., Matsuo M., Tanaka S., Akimoto H., Asahi S., Nishimura S., Katze J.R., Hashizume T., Crain P.F., McCloskey J.A. (1997). Biosynthesis of archaeosine, a novel derivative of 7-deazaguanosine specific to archaeal tRNA, proceeds via a pathway involving base replacement on the tRNA polynucleotide chain. J. Biol. Chem..

[23] Ishitani R., Nureki O., Nameki N., Okada N., Nishimura S., Yokoyama S. (2003). Alternative tertiary structure of tRNA for recognition by a posttranscriptional modification enzyme. Cell.

[24] Hall K.B., Sampson J.R., Uhlenbeck O.C., Redfield A.G. (1989). Structure of an unmodified tRNA molecule. Biochemistry.

[25] Derrick W.B., Horowitz J. (1993). Probing structural differences between native and in vitro transcribed *Escherichia coli* valine transfer RNA: Evidence for stable base modification-dependent conformers. Nucleic Acids Res..

[26] Perret V., Garcia A., Puglisi J., Grosjean H., Ebel J.P., Florentz C., Giege R. (1990). Conformation in solution of yeast tRNA^Asp^ transcripts deprived of modified nucleotides. Biochimie.

[27] Ahn H.J., Kim H.W., Yoon H.J., Lee B.I., Suh S.W., Yang J.K. (2003). Crystal structure of tRNA(m^1^G37)methyltransferase: Insights into tRNA recognition. EMBO J..

[28] Elkins P.A., Watts J.M., Zalacain M., van Thiel A., Vitazka P.R., Redlak M., Andraos-Selim C., Rastinejad F., Holmes W.M. (2003). Insights into catalysis by a knotted TrmD tRNA methyltransferase. J. Mol. Biol..

[29] Ito T., Masuda I., Yoshida K., Goto-Ito S., Sekine S., Suh S.W., Hou Y.M., Yokoyama S. (2015). Structural basis for methyl-donor-dependent and sequence-specific binding to tRNA substrates by knotted methyltransferase TrmD. Proc. Natl. Acad. Sci. USA.

[30] Christian T., Lahoud G., Liu C., Hou Y.M. (2010). Control of catalytic cycle by a pair of analogous tRNA modification enzymes. J.Mol. Biol..

[31] Lahoud G., Goto-Ito S., Yoshida K., Ito T., Yokoyama S., Hou Y.M. (2011). Differentiating analogous tRNA methyltransferases by fragments of the methyl donor. RNA.

